# Low-Dose Naltrexone for Pruritus in Systemic Sclerosis

**DOI:** 10.1155/2011/804296

**Published:** 2011-09-12

**Authors:** Tracy Frech, Kirsten Novak, Monica P. Revelo, Maureen Murtaugh, Boaz Markewitz, Nathan Hatton, Mary Beth Scholand, Edward Frech, David Markewitz, Allen D. Sawitzke

**Affiliations:** ^1^Division of Rheumatology, Department of Internal Medicine, University of Utah, Salt Lake City, UT 84132, USA; ^2^Department of Medicine, Salt Lake City Veterans Affairs Medical Center, Salt Lake City, 84148 UT, USA; ^3^Department of Pathology, University of Utah, Salt Lake City, UT 84112, USA; ^4^Division of Epidemiology, Department of Internal Medicine, University of Utah, Salt Lake City, UT 84132, USA; ^5^Division of Respiratory, Critical Care and Occupational Pulmonary Medicine, Department of Internal Medicine, University of Utah, Salt Lake City, UT 84132, USA; ^6^Mountain West Gastroenterology, Salt Lake City, UT 84107, USA; ^7^University of Utah Scleroderma Center, University of Utah, Salt Lake City, UT 84132, USA

## Abstract

Pruritus is a common symptom in systemic sclerosis (SSc), an autoimmune disease which causes fibrosis and vasculopathy in skin, lung, and gastrointestinal tract (GIT). Unfortunately, pruritus has limited treatment options in this disease. Pilot trials of low-dose naltrexone hydrochloride (LDN) for pruritus, pain, and quality of life (QOL) in other GIT diseases have been successful. In this case series we report three patients that had significant improvement in pruritus and total GIT symptoms as measured by the 10-point faces scale and the University of California Los Angeles Scleroderma Clinical Trials Consortium Gastrointestinal Tract 2.0 (UCLA SCTC GIT 2.0) questionnaire. This small case series suggests LDN may be an effective, highly tolerable, and inexpensive treatment for pruritus and GIT symptoms in SSc.

## 1. Introduction

Systemic sclerosis (SSc; scleroderma) is an autoimmune disease characterized by vasculopathy and fibrosis of multiple organs including the skin, lungs, and gastrointestinal tract (GIT). This chronic disease process results in pain and pruritus, two distinct, but interacting phenomena [1]. Pruritus is most common in the early stages of disease and may subside as the disease progresses [2]. SSc patients that complain of pruritus have more significant skin involvement, more severe finger ulcers, worse respiratory symptoms, and a greater number of GIT complaints [3]. Of interest, pruritus is independently associated with GIT symptoms in SSc [3]. Although pruritus is associated with significant disability, management guidelines for pruritus in SSc do not exist [4]. 

Pruritus is also a feature of primary biliary cirrhosis (PBC), which occurs more commonly in SSc than the normal population [5]. There is a known association with PBC and oxidative stress as well as endothelial dysfunction [6]. Pharmaceutical management suggestions for treatment of pruritus in PBC include cholestyramine, rifampin, sertraline, and naloxone [7]. More recently, pilot trials of low-dose naltrexone hydrochloride (LDN), which is a pharmaceutical similar to naloxone, have recently gained increasing recognition for treating chronic pain associated with fibromyalgia, multiple sclerosis, and Crohn's disease [8–10]. 

Evidence supporting the hypotheses that increased opioid-mediated neurotransmission in the brain is a mechanism of pruritus and that central opioidergic tone is increased in cholestasis provides a rationale for treating the pruritus of cholestasis with opiate antagonists in PBC [11]. Another potential mechanism of action of LDN is through attenuation of the production of proinflammatory cytokines and superoxides potentially mediated by activity of toll-like receptor 4 [12]. Modulation of mitochondrial apoptosis has also been proposed as a mechanism of LDN [13]. In SSc, oxidative stress may be important in disease pathogenesis [13]. Thus, an agent that potentially modulates oxidative stress is attractive as an emerging therapeutic in SSc. Given the putative mechanisms of action of LDN and the roles of these various pathways in SSc, our hypothesis is that LDN may be a reasonable agent to treat pruritus in SSc.

## 2. Patients and Methods

In this case series we report three SSc patients who presented to the University of Utah SSc Center with the chief complaint of pruritus. All were white females between the ages of 34 and 56. Two patients were of diffuse cutaneous SSc subtype (dSSc); the other one was limited SSc subtype (lSSc) [14]. One of the dSSc patients and the lSSc had tendon friction rubs. None had scleredema. The disease duration from the first non-Raynaud's phenomenon symptom varied from 1 year to 11 years. Raynaud's phenomenon onset was within the same year of diagnosis in two patients, and three years prior in one patient. All patients reported pruritus as an important feature of disease since onset of SSc and did not attribute it directly to change in skin thickening or GI manifestations. They all reported that “itchiness” of the skin had been present for >6 weeks and was unresponsive to antihistamines on all days. None had urticaria. They all denied a history of eczema or skin conditions other than SSc, any new product, or infectious exposures and attributed this symptom to SSc. A ten-point faces scale was used to assess pruritus. On this scale 0, is no symptoms, 1 very mild, 2 discomforting, 3 tolerable, 4 distressing, 5 very distressing, 6 intense, 7 very intense, 8 utterly horrible, 9 excruciating, 10 unimaginable/unspeakable [15]. It has been used in other clinical trials of pruritus [16]. Each of our participants ranked their pruritus >5. 

All patients completed a University of Los Angeles Scleroderma Clinical Trials Consortium Gastrointestinal Tract Questionnaire (GIT 2.0). The GIT 2.0 is patient-reported outcome measure to assess health-related quality of life (HRQOL) and GIT severity in SSc [17, 18]. This 34-item instrument has seven scales: reflux, distention/bloating, diarrhea, fecal soilage, constipation, emotional well-being, and social functioning and a total GI score (a higher score denoted worse HRQOL). All scales are scored from 0.0 (better HRQOL) to 3.0 (worse HRQOL) except diarrhea and constipation scales that range from 0.0 to 2.0 and 0.0 to 2.5, respectively. The total GI score is the average of 6 of 7 scales (excludes constipation), and total GI score is scored from 0.0 (better HRQOL) to 3.0 (worse HRQOL). Each scale is further divided into 3 groups by severity: none-to-mild, moderate, and severe-to-very severe. These were calculated based on anchors included in the original published data and data collected in a recent National Scleroderma Foundation online survey and available online at http://uclascleroderma.researchcore.org. The instrument takes 6–8 minutes to be completed and has been shown to have acceptable reliability and validity [17, 19]. Additionally each patient had a modified Rodnan skin score (mRSS) as part of their routine care at baseline (Table 1). 

**Figure 1:**
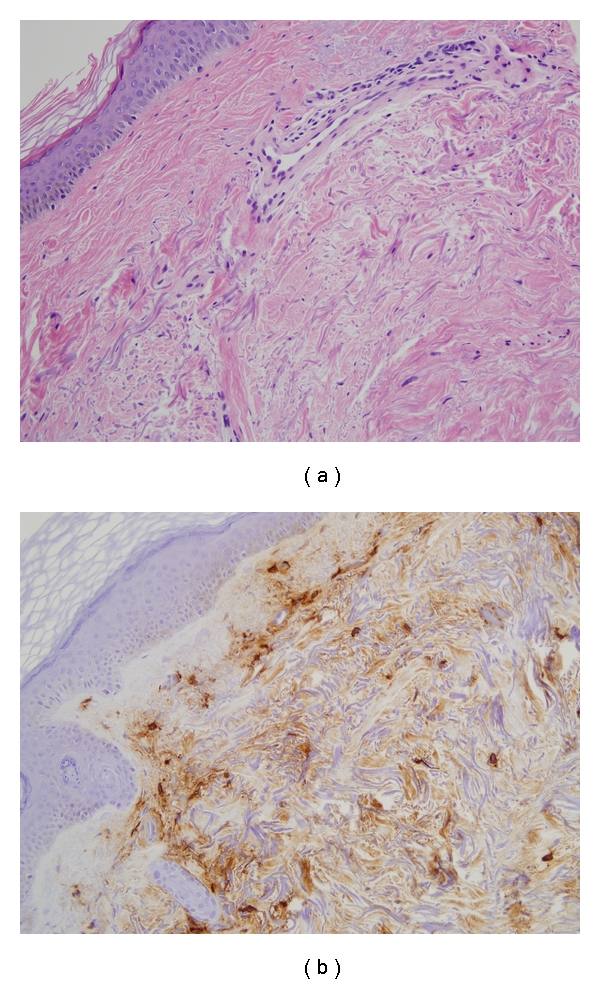
Biopsy specimens from patient 1: H&E-stained section shows moderate perivascular and periadnexal inflammation and thickening of dermal collagen (20X). There are several mast cells in perivascular, interstitial, and periadnexal distribution with granular-appearing cytoplasm (IHC mast cell tryptase 20X).

**Figure 2:**
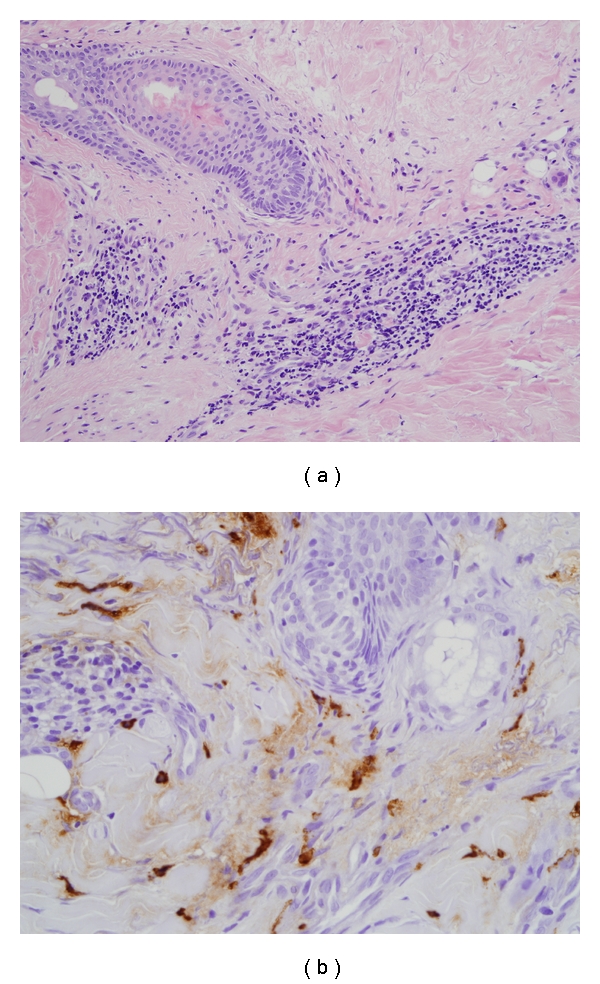
Biopsy specimens from patient 3: H&E-stained section shows scant perivascular inflammation and thickening of dermal collagen (20X). There are scattered mast cells in perivascular and interstitial distribution with granular-appearing cytoplasm (IHC mast cell tryptase 20X).

Baseline laboratories were obtained in all patients as per routine care. All were antinuclear antibody (ANA) positive. A marker of inflammation (C-reactive protein) was normal in all patients. None of the patients had an eosinophilia (<0.4 k/*μ*L) on peripheral blood smear. Anti-tissue transglutaminase and antimitochondrial antibody were negative in all patients. Two patients had skin biopsy performed as part of another clinical study. A computer-assisted image analysis of two of the biopsy specimens revealed that at baseline there was not an increase in mast cells greater than those present in a healthy control (Figures 1 and 2). 

From a treatment perspective, all patients were RNA polymerase III positive, making a trial of corticosteroids for treatment of pruritus an unattractive option due to an increase risk of scleroderma renal crisis [20]. They all denied depression and were resistant to a trial of sertraline. None consumed alcohol or were taking narcotics for pain. One patient was on methotrexate for arthritis. The other two patients were not on immunosuppression medications. All patients denied respiratory complaints, had no evidence of pulmonary arterial hypertension on echocardiogram, and had normal pulmonary function tests. No patients had digital ulcers or met clinical criteria for fibromyalgia. All had normal colonoscopies without evidence of collagenous colitis.

Each patient was initiated on LDN 2 mg by mouth at bedtime for the first month. Using dosing recommendations from another trial and phone call monitoring for adverse effects, the dose was increased by 1 mg by mouth at bedtime each week (0.5 mg the final week) up to a maximum dose of 4.5 mg [9]. One patient requested to maintain the dose of 2 mg by mouth at bedtime, as she felt it was working well and did not wish to incur additional cost. The other two patients reached the 4.5 mg dose. All patients were seen in clinic for followup after two months of treatment. All patients repeated the GIT 2.0, and a mRSS was done at each visit. No other medication change, including use of over-the-counter agents, was allowed for any of these patients during the treatment period. However, nonmedicinal changes were allowed. One patient implemented head-of-the-bed elevation for reflux management, and two patients increased fluid and dietary fiber intake for symptoms of constipation.

## 3. Results

All patients reported an improvement in pruritus on a Faces Scale after initiating LDN (Table 1) [15]. In two different patients, pruritus completely resolved on LDN. In two patients a trend in improvement in mRSS was seen at two months, which is not clinically significant [21]. However, all patients reported feeling that skin was objectively softer. In all patients there was an improvement in total GIT 2.0 scores as well as in constipation and distention/bloating subscales. Repeat skin biopsy specimens were not obtained. No adverse drug effects were reported with the exception of two nights of insomnia reported in a single patient.

## 4. Discussion

Pruritus is a common symptom in SSc, with limited treatment options. The pathogenesis of chronic (>6 weeks duration) pruritus is complex and involves a network of resident cells (such as mast cells, keratinocytes, and sensory neurons) and transient inflammatory cells within the skin [22]. Pruritus in SSc is independently associated with GI symptoms [3]. Because of the association of pruritus with quality of life (QOL) in SSc, more attention to potential methods for intervention is warranted [23]. This small case series suggests a potential role for LDN for SSc with pruritus. Additionally, a possible improvement in mRSS and in GIT symptoms may also result from use of LDN. 

 Autoimmune gastrointestinal disorders such as inflammatory bowel disease (IBD), gluten-sensitive enteropathy, and PBC have also been associated with pruritus [24]. Evidence supports that increased opioid-mediated neurotransmission in the brain may be a mechanism of pruritus in PBC [11]. LDN has been found to improve QOL in IBD [10]. Although the real biological mechanism of LDN is not known, LDN may modulate pruritus through opioid-mediated actions or a reduction of inflammatory mediators [25, 26]. As such, a better understanding of the interaction of the GIT and pruritus is warranted. 

SSc patients commonly have diarrhea, constipation, and distention/bloating that may mimic GIT hypersensitivity [27]. Whether the GIT is responsible for the pruritus in SSc or whether the GIT and skin are reacting to a similar stimulus or endogenous immune response is unknown. Mast cells have been suggested to have an important role in SSc as well as in functional bowel disorders [28, 29]. However, computer-assisted analysis of skin biopsy specimens in two of our patients did not reveal an abnormal percentage of mast cells. 

Clearly a larger number of patients and a double-blind placebo-controlled trial are needed to define the therapeutic potential of LDN in SSc. The patients' subjective observation of “softer skin” was not validated by a significant change in mRSS and could be attributed to the natural history of disease rather than to naltrexone. Additionally, intraobserver variability could explain the improvement in mRSS. Psychological and physiological predictors of pruritus response as well as minimally important differences in the ten-point faces scale need to be defined. Gastrointestinal biopsy specimens would have been helpful for correlating to skin biopsy specimens for defining potential morphologic and histochemical change. Nonetheless, this series suggests LDN may be an effective, highly tolerable, and inexpensive treatment for pruritus in SSc and further supports a potential role for computer-assisted quantification of inflammatory cell types in skin biopsies to guide selection of therapeutics.

## Figures and Tables

**Table 1:** Clinical features of systemic sclerosis participants.

Patient	Disease onset*, subtype	Pre-LDN pruritus	Post-LDN pruritus	Pre-LDN mRSS	Post-LDN mRSS	Pre-LDN UCLA SCTC GIT 2.0	Post-LDN UCLA SCTC GIT 2.0
1	2 years, diffuse	10	4	28	24	Total: 0.355 (i) Reflux: 0.63 (ii) Distention: 0.5 (iii) Soilage: 0 (iv) Diarrhea: 0 (v) Constipation: 1.25 (vi) Social: 0 (vii) Emotional: 0.11	Total: 0.302 (i) Reflux: 0.5 (ii) Distention: 0.5 (iii) Soilage: 0 (iv) Diarrhea: 0 (v) Constipation: 1 (vi) Social: 0 (vii) Emotional: 0.11

2	11 years, diffuse	6	0	19	17	Total: 0.395 (i) Reflux: 0.63 (ii) Distention: 1.75 (iii) Soilage: 0 (iv) Diarrhea: 0.5 (v) Constipation: 0.5 (vi) Social: 0.17 (vii) Emotional 0.22	Total: 0.216 (i) Reflux: 0.38 (ii) Distention: 0.5 (iii) Soilage: 0 (iv) Diarrhea: 0 (v) Constipation: 0.25 (vi) Social: 0.17 (vii) Emotional 0.22

3	1 year, limited	8	0	12	12	Total: 0.54 (i) Reflux: 0.25 (ii) Distention: 1.5 (iii) Soilage: 0 (iv) Diarrhea: 0 (v) Constipation: 1.25 (vi) Social: 0.33 (vii) Emotional 0.44	Total: 0.216 (i) Reflux: 0.13 (ii) Distention: 0.5 (iii) Soilage: 0 (iv) Diarrhea: 0 (v) Constipation: 0.5 (vi) Social: 0.17 (vii) Emotional 0.22

*First non-Raynaud's phenomenon symptom; LDN: low-dose naltrexone; mRSS: modified Rodnan skin score; UCLA SCTC GIT 2.0: University of California Los Angeles Scleroderma Clinical Trials Consortium Gastrointestinal Tract 2.0.
